# Surgical extent as a determinant of systemic reserve after lung resection: Why spirometric recovery does not capture the true physiological cost

**DOI:** 10.1016/j.xjon.2026.101854

**Published:** 2026-05-06

**Authors:** Ichiro Yoshino, Hisashi Saji

**Affiliations:** a Department of Thoracic Surgery, International University of Health and Welfare Narita Hospital, Narita, Japan; b Department of Thoracic Surgery, St Marianna University, Kawasaki, Japan

**Keywords:** non–small cell lung cancer, segmentectomy, sarcopenia, surgical metabolism

**Figure:**
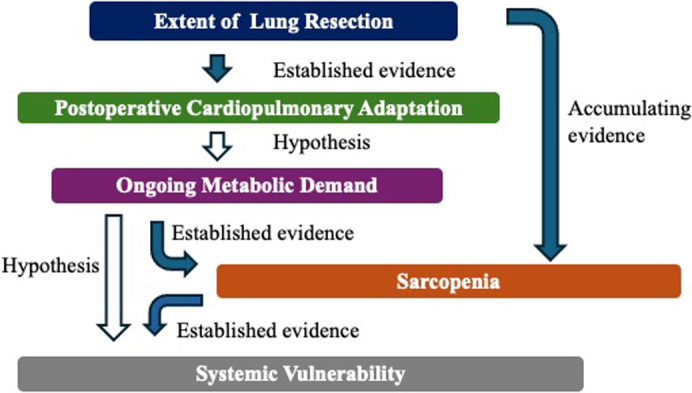
Conceptual framework linking extent of lung resection to systemic resilience.

**Central Message**

In lung surgery, the metabolic cost associated with postoperative adaptation may be manifested as sarcopenia, and the cost could be a trade-off with oncologic radicality in selection of procedure.

## Introduction: The Unsolved Question After JCOG0802

For thoracic surgeons, one of the most important unresolved questions after the JCOG0802/WJOG4607L randomized trial is why overall survival differs between segmentectomy and lobectomy despite similar postoperative spirometric recovery. The trial confirmed a superior overall survival profile for segmentectomy in small peripheral non–small cell lung cancer. Notably, this advantage was driven less by oncologic outcomes and more by a reduction in non-lung cancer–related mortality.^1^ Indeed, high nonprimary disease mortality was observed in the lobectomy group. Remarkably, although the occurrence of secondary cancers was similar across groups, deaths from these cancers remained more frequent in the lobectomy group.^1^ Although these findings have had substantial clinical impact, they have also generated uncertainty and debate. In the absence of a readily apparent oncologic or functional explanation, some have questioned whether the survival difference reflects chance, unmeasured confounding, or trial-specific effects. This skepticism has been further amplified by the recently presented 10-year follow-up data at the 105th Annual Meeting of The American Association for Thoracic Surgery.^2^ In that analysis, the overall survival curve continued to numerically favor segmentectomy, yet the hazard ratio approached 1.0. Select commentaries have interpreted these findings as evidence favoring lobectomy, underscoring the challenges of interpreting long-term survival trends in aging cohorts.^3^

**Figure 1:**
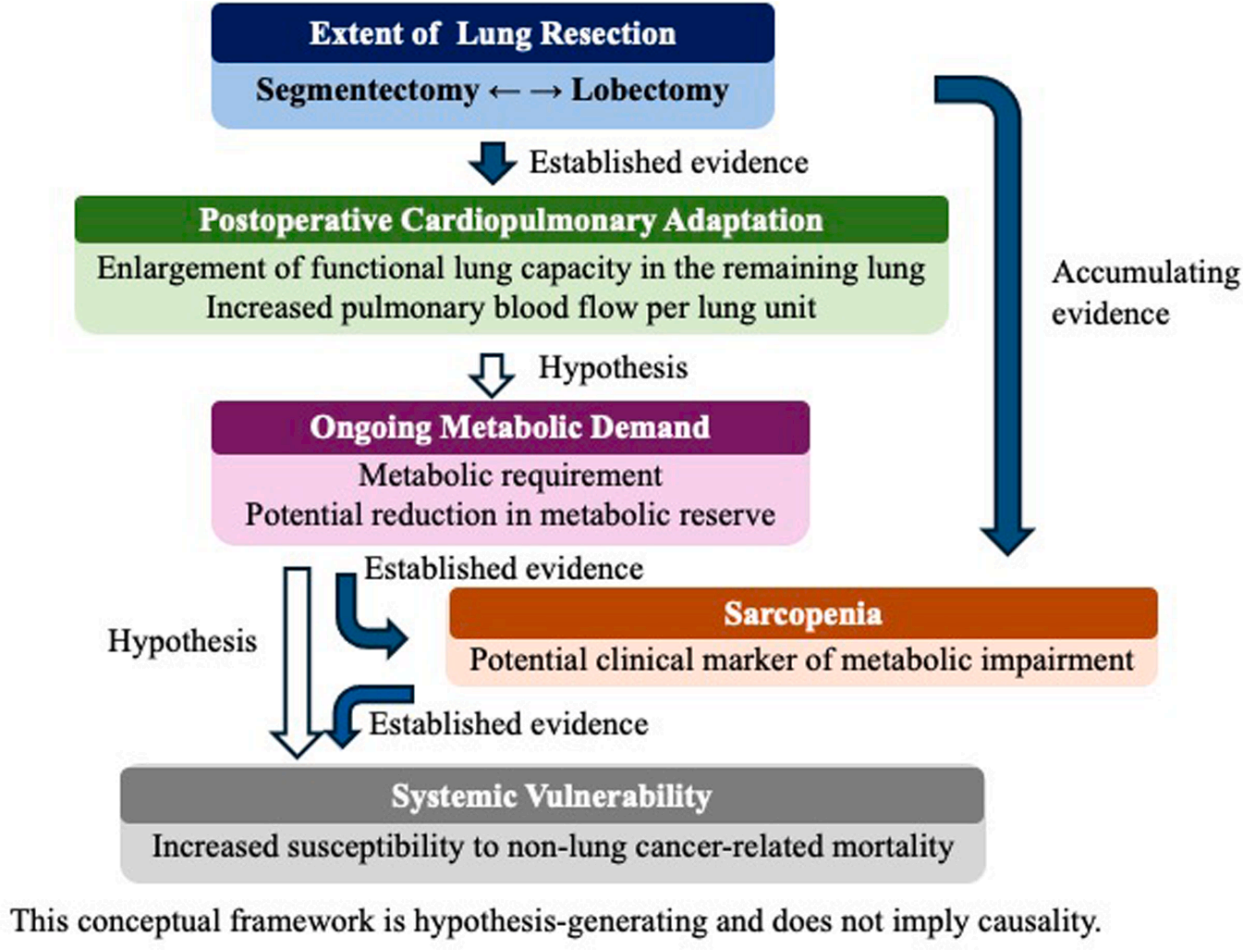
Conceptual framework linking extent of lung resection to systemic resilience. Hypothesis-generating framework illustrating how the extent of lung resection may influence long-term survival through differences in postoperative physiological adaptation.

This finding fundamentally challenges traditional assumptions regarding the extent of resection. Conventional interpretations have emphasized oncologic radicality and postoperative pulmonary function as primary determinants of long-term outcome. However, if pulmonary function recovers to a similar degree after different extents of resection, then alternative physiological mechanisms must be considered to explain the persistent survival difference observed years after surgery. This discrepancy necessitates a shift in focus toward alternative physiological mechanisms. We must consider how the initial surgical trauma and the permanent loss of lung parenchyma trigger chronic systemic stress, which influences long-term survival independently of traditional respiratory metrics (Figure 1).

## Spirometric Recovery and the Limits of Functional Metrics

Postoperative pulmonary function, primarily forced expiratory volume in 1 second and vital capacity, has long served as the principal marker of surgical impact. In the JCOG0802 trial, however, differences in spirometric parameters between segmentectomy and lobectomy remained relatively modest beyond the early postoperative period.^1^ This apparent equivalence is often misinterpreted as evidence that the physiological burden of lobectomy is merely transient. Recent evidence suggests an alternative interpretation. A systematic meta-analysis demonstrated that postoperative function at 1 year recovers proportionally to the resection extent, reflecting the remaining lung's substantial compensatory capacity.^4^ Thus, similar spirometric values may represent successful physiological compensation rather than equivalent biological states.

Critically, restoring measured lung volumes is not metabolically neutral. Comparable pulmonary performance is often achieved through sustained cardiopulmonary adaptation—encompassing both morphologic remodeling and functional augmentation—which imposes a persistent physiological workload. Classical studies confirm that surgical stress induces prolonged neuroendocrine and cardiopulmonary responses, increasing basal metabolic demands well beyond the acute phase.^5^ Consequently, limited spirometric differences in JCOG0802 do not negate long-term stress; instead, they suggest that patients undergoing extensive resection maintain function by paying a hidden physiological cost uncaptured by conventional metrics.

Many studies have shown lung resection causes persistent stress on the pulmonary circulation and right heart. Beyond local effects, this compensatory local response influences systemic metabolism via interorgan signaling network.^6^ Consequently, skeletal muscle conditions like sarcopenia may serve as key surrogates for this metabolic crosstalk after surgery, and this “lung-muscle metabolic axis” framework is described in the sections that follow.^7^

## The Framework of Postlung Resection Change in Physiological Status

Preoperative skeletal muscle quantity and quality are powerful, independent predictors of survival in patients with lung cancer who undergo surgery.^8^ These findings underscore systemic physiological reserve as a key determinant of postoperative resilience. Muscle tissue supplies energy (adenosine triphosphate) and nutrients (amino acids, glycogen, fatty acids, etc) to various organs through an interorgan network mediated by humoral factors such as myokines or cytokines and hormones.^9^ In this context, loss of skeletal muscle mass may be viewed as an integrative biomarker of long-term physiological burden. It captures the cumulative effects of chronic stress, metabolic demand, and impaired somatic maintenance, all of which are poorly reflected by traditional cardiopulmonary metrics.

It has also become clear that the progressive loss of skeletal muscle mass after surgery also has a strong impact on prognosis, and several studies indicate the decrease is related to deterioration of respiratory function or of nutrition status.^10^^,^^11^ Recent longitudinal evidence suggests that the extent of lung resection itself influences the postoperative skeletal muscle trajectories.^12^ This observation provides a critical link between operative strategy and systemic metabolic outcomes. Surgical extent acts as a key determinant of physiological economy rather than a simple anatomical measure. Significant parenchymal loss triggers a compensatory burden that accelerates the exhaustion of systemic physiological reserves. This framework integrates metabolic trade-offs in chronic physiological adaptation.^7^ Proliferative remodeling during lung expansion demand ongoing energy and nutrient investment proportional to parenchymal loss.^13^^,^^14^ The cardiopulmonary system must continuously operate under altered hemodynamics—increased pulmonary blood flow, right ventricular workload, and sympathetic activation—to maintain oxygen delivery.^15^ Consequently, these adaptive processes impose a persistent metabolic burden that may divert resources from systemic physiological maintenance.

From a systems perspective, such chronic investment in functional maintenance inevitably competes with energy allocation to other somatic processes, including skeletal muscle preservation, immune competence and tissue repair, that is observed in major surgeries in general.^16^^,^^17^ Under conditions of such a chronic stress, energy invested in immediate functional maintenance may come at the expense of long-term somatic maintenance.^18^^,^^19^ Within this framework, postoperative sarcopenia may represent a downstream manifestation of such a chronic energy reallocation. This interpretation reconciles preserved spirometric function with declining systemic physiological reserve and provides a unifying explanation for the observed associations among surgical extent, muscle loss, and non-lung cancer–related mortality.

## Reinterpreting the JCOG0802 Paradox

The JCOG0802 trial's core debate centers on oncologic validity versus functional preservation. However, its most striking finding—reduced non-lung cancer mortality after segmentectomy—suggests the surgical scope has a broader systemic impact. Despite similar incidence rates, lower mortality from other cancers indicates enhanced treatment resilience. Following the model proposed by Williams and colleagues,^20^ skeletal muscle mass represents a metabolic reserve; its depletion by age, malignancy and treatment creates systemic vulnerability. While cardiovascular mortality remained unchanged—likely due to stringent patient selection—postresection survival for patients with Japanese stage IA2 (88%) was notably lower than the JCOG lobectomy cohort, even when including low-grade tumors (C/T < 0.5).^21^ The reason why such a trade-off result showed the superiority of segmenetctomy, which has a low metabolic cost, is because the trial was conducted on lung cancer, which has a relatively lower biological risk (good life prognosis), and in this respect it can be reasonably explained as being different from the CALGB140503 trial.^22^ In JCOG0802's high-survival cohort (≥90%), subtle physiologic benefits of lung preservation likely emerged. Conversely, the lower survival (60-70%) in CALGB140503 may have masked these effects due to high competitive mortality. Although differing lymph node management and recurrence definitions hinder direct comparison, the systemic benefits of lung preservation should apply to all patients—provided oncologic objectives are met. Viewed through this lens, the paradox might be redefined: Parenchymal preservation lowers metabolic demand and improves survival, whereas spirometry captures only a partial physiologic picture.

## Implications for Surgical Strategy

These considerations have important implications. If preoperative muscle status predicts survival and postoperative loss depends on surgical extent, preserving physiological reserve is a core surgical objective. For selected patients with early-stage non–small cell lung cancer, sublobar resections may confer survival benefits by ensuring oncologic adequacy, preserving pulmonary function, and mitigating the long-term burden of chronic adaptation. Surgical extent is therefore a long-term risk factor influencing systemic resilience, not merely a technical choice.

Physical activity is known to improve cancer prognosis,^23^^,^^24^ highlighting the importance of maintaining muscle mass and strength within our framework. Implementing multidisciplinary rehabilitation—encompassing exercise, nutrition, and respiratory care—from the perioperative phase through long-term recovery can bolster systemic reserve and alleviate the metabolic burden of sustained cardiopulmonary adaptation.

## Future Perspectives

We hope this framework will open up new horizons for clinical practice and research in thoracic surgery. Longitudinal studies are needed to evaluate the correlation between resected lung volume and integrated physiological markers, including skeletal muscle mass status, pulmonary arterial stress including pulmonary artery/aorta ratio,^25^ echocardiographic hemodynamics, and metabolic expenditure (basal metabolic rate and total energy expenditure).^18^ Integrating wearable monitoring, and metabolomic profiling will allow for a more precise quantification of systemic physiological load.^E1^ Additionally, investigating humoral factors such as angiotensin II, natriuretic peptides, growth differentiation factor 15, and myokines will be vital to advancing our understanding of this interorgan network.^E2^

In addition, interventional studies targeting postoperative metabolic support, exercise, and nutritional optimization may clarify whether preservation of skeletal muscle can modify long-term outcomes after lung resection. Such approaches may ultimately shift the focus of thoracic surgery from purely anatomical preservation to systemic physiological optimization.

## Conclusions

The extent of lung resection influences chronic cardiopulmonary adaptation and energy allocation, potentially affecting systemic resilience (Figure 1). By preserving physiological reserve, sublobar resection can help reduce non-lung cancer mortality. In this era of precision surgery, surgical extent should be recognized as a meaningful determinant of lifelong physiological economy, standing alongside anatomical radicality. Balancing oncological goals with functional preservation remains a key consideration in modern thoracic care.

## Conflict of Interest Statement

I.Y. reported honoraria for lectures from AstraZeneca, Chugai Pharmaceutical, Johnson & Johnson, Covidien Japan, Medicaroid, Intuitive Surgical, and MSD, but these are not related to the present paper. H.S. reported no conflicts of interest.

The *Journal* policy requires editors and reviewers to disclose conflicts of interest and to decline handling or reviewing manuscripts for which they may have a conflict of interest. The editors and reviewers of this article have no conflicts of interest.
